# Prognostic factors of poor outcome in patients with upper limb replantation surgery admitted to the icu

**DOI:** 10.1186/2197-425X-3-S1-A235

**Published:** 2015-10-01

**Authors:** E Portugal Rodríguez, E Martínez Barrio, E Nevado Sánchez, A Rodríguez Vega, A Berrazueta Sánchez de Vega, D Armesto Formoso, A Zabalegui Pérez, M Martínez Barrios, JA Fernández Ratero, S Puerto Corrales

**Affiliations:** Burgos University Hospital, Burgos, Spain

## Intr

Our University Hospital is one of the national reference centers in upper extremity replantation surgery (ERS) (the transfer is carried out on air transportation by the National Transplant Organization, NTO). Therefore, it is important to optimize postoperative care in the ICU that may influence replantation evolution.

## Objectives

To describe the characteristics of patients undergoing this surgery admitted to the ICU and to evaluate prognostic factors related with bad outcome [2].

## Methods

Observational study of patients with ERS during a period of seven years (2008-2014). Computer research of electronic medical records in databases was conducted. We obtained a sample of 60 cases and classified them into two groups: good and poor outcome. We analyzed: epidemiological variables (age, smoking, diabetes, arteriopathy) variables related with injury mechanism (degloving, flattening, large bone lesion, neural lost and vascular affectation), surgery and postoperative care (amines, mechanical ventilation, transfusion, ICU stay).

We compared the factors associated with poor outcome in both groups

## Results

In our sample of 60 cases 85% were male, mean age was 47,13 years, and no differences in previous comorbidities were observed. We found significant differences between the injury mechanism and the presence of large bone and vascular lesion (Table 1). Time of ischemia and surgery were comparable. There were no treatment differences in the ICU in both groups. ICU replantation survival was 93,3%, with 10% of early reoperation due to arterial thrombosis.

**Table 1:** Table 1

Variable	Good outcome (n = 30)	Bad outcome (amputation, n = 30)	P value
Injury mechanism(%) (degloving, flattening)	34.8	65.2	0.09
Large bone lesion(%)	21.7	78.3	0.00
Large vascular affectation (%)	18.8	81.3	0.00
Large neural lost(%)	44.7	55.3	0.11
Warm ischemia time (mean hours ± SD)	4,0 ± 1,44	4.36 ± 1.45	0.57
Surgery time (median hours, IQR)	4.00 (3,15-7,50)	5.00 (3.00-8.30)	0.61

## Conclusions

In our sample, large bone and vascular affectation were the factors associated with poor replantation outcome, in patients with upper extremity amputation. No differences related with surgical or postoperative care variables were observed. Replantation survival rates, and need of reoperation in our series, were consistent with literature data [3].
